# COX-2-PGE_2_-EPs in gynecological cancers

**DOI:** 10.1007/s00404-020-05559-6

**Published:** 2020-05-03

**Authors:** Yao Ye, Xipeng Wang, Udo Jeschke, Viktoria von Schönfeldt

**Affiliations:** 1 Department of Gynecology and Obstetrics, Xin Hua Hospital, Shanghai Jiao Tong University School of Medicine, 1665, Kongjiang Road, Yangpu District, Shanghai, 200000 People’s Republic of China; 2 Department of Obstetrics and Gynecology, Ludwig-Maximilians University of Munich, Campus Großhadern: Marchioninistraße 15, 81377 Munich, Germany; 3 University Hospital Augsburg, Augsburg, Germany

**Keywords:** Cyclooxygenase-2 (COX-2), Prostaglandin E_2_ receptors (EPs), Endometrial cancer, Ovarian cancer, Cervical cancer

## Abstract

**Purpose:**

Nonsteroidal anti-inflammatory drugs (NSAIDs) and selective COX-2 inhibitors (COXibs) inhibit the progression of endometrial cancer, ovarian cancer and cervical cancer. However, concerning the adverse effects of NSAIDs and COXibs, it is still urgent and necessary to explore novel and specific anti-inflammation targets for potential chemoprevention. The signaling of cyclooxygenase 2-prostaglandin E_2_-prostaglandin E_2_ receptors (COX-2-PGE_2_-EPs) is the central inflammatory pathway involved in the gynecological carcinogenesis.

**Methods:**

Literature searches were performed to the function of COX-2-PGE_2_-EPs in gynecological malignancies.

**Results:**

This review provides an overview of the current knowledge of COX-2-PGE_2_-EPs signaling in endometrial cancer, ovarian cancer and cervical cancer. Many studies demonstrated the upregulated expression of the whole signaling pathway in gynecological malignancies and some focused on the function of COX-2 and cAMP-linked EP2/EP4 and EP3 signaling pathway in gynecological cancer. By contrast, roles of EP1 and the exact pathological mechanisms have not been completely clarified. The studies concerning EP receptors in gynecological cancers highlight the potential advantage of combining COX enzyme inhibitors with EP receptor antagonists as therapeutic agents in gynecological cancers.

**Conclusion:**

EPs represent promising anti-inflammation biomarkers for gynecological cancer and may be novel treatment targets in the near future.

## Introduction

**Fig. 1:**
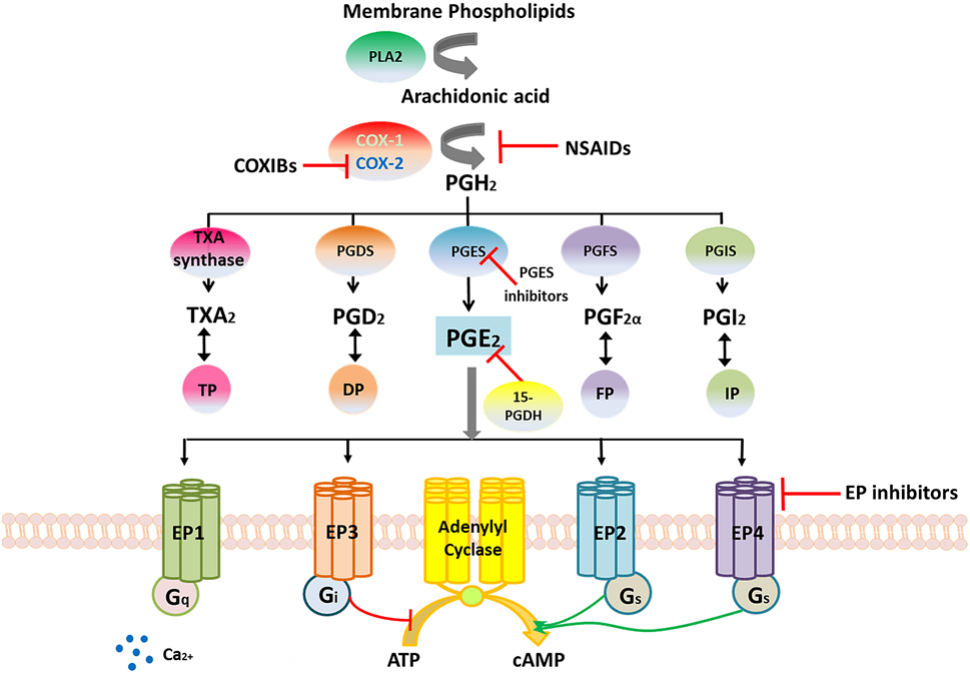
COX-2-PGE_2_-EPs signaling pathway. Arachidonic acid is released from the membrane phospholipids by PLA2 and then is metabolized by COX-1 and COX-2 into PGH_2_. PGH_2_ is converted by specific isomerases (PGDS, PGES, PGFS and PGIS) and TXA synthase to multiple prostaglandins (PGE_2_, PGD_2_, PGF_2α_, PGI_2_) and the thromboxane A_2_ [4]. Prostaglandins act through relative receptors (EP, DP, FP, IP and TP) to mediate their effects [5]. The inhibitors of COX-2-PGE_2_-EPs signaling pathway include nonsteroidal anti-inflammatory drugs (NSAIDs), COX-2 selective inhibitors (COXIBs), PGES inhibitor, 15-hydroxyprostaglandin dehydrogenase (15-PGDH) and EP inhibitors. NSAIDs inhibit the function of both COX-1 and COX-2 while COXIBs only inhibit the function of COX-2. PGE_2_ is degraded by 15-PGDH into an inactive 15-keto PGE_2_ after binding to EP receptors [6]. Both PGES inhibitors and EP inhibitors are novel inhibitors that have been investigating in these years. *PLA2* phospholipases A2, *COX-1* cyclooxygenase-1 *COX-2* cyclooxygenase-2, *PGDS* prostaglandin D synthase, *PGES* prostaglandin G synthase, *PGFS* prostaglandin F synthase, *PGIS* prostaglandin I synthase, *PG* prostaglandin, *EP* prostaglandin E receptor, *DP1.2* prostaglandin D receptor 1.2, *FP* prostaglandin F receptor, *IP* prostaglandin I receptor, *ATP* adenosine triphosphate, *cAMP* cyclic adenosine monophosphate

Abundant literature has demonstrated a strong correlation between chronic inflammation and cancer development since chronic inflammation contributes to the development of over 15% of malignancies worldwide [1]. Plenty of pro-inflammatory factors mediate a role in carcinogenesis, such as tumour necrosis factor (TNF), interleukin (IL)-1α, IL-1β, IL-6, IL-8, IL-18, matrix metallopeptidase-9 (MMP-9), vascular endothelial growth factor (VEGF), cyclooxygenase 2 (COX-2), and arachidonate 5-lipoxygenase (5-LOX) [2]. Serum levels of C-reactive protein (CRP), IL-6, and IL-1 receptor antagonist (IL-1Ra) are significantly associated with endometrial cancer risk when analyzing 246,000 women in ten European countries [3]. The signaling of cyclooxygenase 2-prostaglandin E_2_-prostaglandin E_2_ receptors (COX-2-PGE_2_-EPs) is the central inflammatory pathway involved in the carcinogenesis (Fig. 1). Based on the current information, we aimed to supplement some additional knowledge of COX-2-PGE_2_-EPs in the carcinogenesis of gynecological cancer from the perspective of inflammation.

## Biogenesis and signaling: COX-2-PGE_2_-EPs

Arachidonic acid is released from the membrane phospholipids by phospholipase A2 (PLA2) and then metabolized by the enzyme of COX-1 and COX-2 into prostaglandin H_2_ (PGH_2_). PGH_2_ is converted by specific isomerases (PGDS, PGES, PGFS and PGIS) and TXA synthase to various prostaglandins (PGE_2_, PGD_2_, PGF_2α_, PGI_2_) and the thromboxane A_2_ (TxA_2_) [4] (Fig. 1). All these prostaglandins (PGE_2_, PGD_2_, PGF_2α_, PGI_2_ and TXA_2_) act through relative specific G-protein coupled receptors (GPCR) to mediate their effects, referred to as the EP, DP, FP, IP and TP receptors [5] (Fig. 1).

COX enzymes are the primary enzymes in the synthesis of eicosanoids and exist in two isoforms: COX-1 is considered to be ubiquitously expressed [7], whereas COX-2 is expressed predominantly in inflammatory cells and upregulated in chronic and acute inflammations [8]. COX-1 and COX-2 are located on human chromosomes 9 and 1 respectively [9]. PGs produced by COX-1 are crucial for maintaining the integrity of gastric mucosa, normal platelet aggregation and renal function, while PGs derived by COX-2 contributes to cancer progression and metastasis [10]. The COX-2 expression is stimulated by different growth factors, cytokines and prostaglandins, which is associated with inflammatory response and is seen as a prognostic factor for malignancy [11, 12]. Furthermore, upregulation of COX-2 and PGE_2_ has been identified in many human cancers and precancerous lesions, and COX inhibitory drugs show protective effects in colorectal cancer and breast cancer [13].

The three distinct synthases contributing to PGE_2_ synthesis are consist of microsomal PGE synthase-1 (mPGES-1), mPGES-2 and cytosolic PGE synthase (cPGES) [14, 15]. There are two separate PGE_2_-biosynthetic routes: the cPLA2-COX-1-cPGES and cPLA2-COX-2-mPGES pathways [15]. COX-2 linked to mPGES is essential for delayed PGE_2_ biosynthesis, which may be linked to inflammation, fever, osteogenesis, and cancer [15]. mPGES-1 is primarily responsible for increasing PGE_2_ levels during inflammation and carcinogenesis, and elevated levels of mPGES-1 present in a number of human cancers, such as colon, lung, stomach, pancreas, cervix, prostate and head and neck squamous carcinoma [16].

PGE_2_ is the most abundant prostaglandin in humans and is known as a key mediator in inflammation. The functions of PGE_2_ are mainly facilitated by specific membrane-bound G-protein-coupled EP receptors (EP1-EP4) with various signaling pathways. EP1 is coupled to the G protein alpha q (G_q_) to mobilize intracellular Ca^2+^, EP2 and EP4 are coupled to the G protein alpha stimulator (G_s_) to activate adenylyl cyclase (AC), and EP3 is mainly coupled to the G protein alpha inhibitor (G_i_) to suppress AC [17]. The EP3 receptor can also be coupled to G_12/13_ proteins, resulting in the activation of the small G protein Rho [18]. After binding its receptor, PGE_2_ can be catalyzed by 15-hydroxyprostaglandin dehydrogenase (15-PGDH) into an inactive 15-keto PGE_2_ [6].

In cancer development, EP1 mediates tumor cell migration, invasion and adjustment to hypoxia environment; EP2 induces angiogenesis and suppresses the anti-tumor immune response; EP4 can mediate tumor cell migration, metastasis, as well as promote aberrant DNA methylation [18]. The role of EP3 in carcinogenesis is still unclear with conflicting effects in distinct cancer cells. EP3 is a unique PGE_2_ receptor, since the human EP3 gene consists of ten exons and nine introns, encoding at least eight distinct EP3 splice variants [19]. EP3 isoforms differ in the amino acid sequences in their specific C-terminal tails and signal transduction pathways by activating different second messengers [20, 21]. This might increase the complexity of investigating the effects of EP3 on the pathological mechanism of cancer development. Studies concerning the COX-2-PGE_2_-EPs expression have been investigated recently and are summarized in this review.

## Crosstalks with other signaling pathways in cancer

Wang et al. elucidated crosstalks interacting with COX-2-PGE_2_-EPs signaling pathways in carcinogenesis, mainly consisting of the epidermal growth factor receptor (EGFR) pathway, nuclear receptor pathway, and Ras-mitogen-activated protein kinase cascade (Ras-MAPK) pathway [7]. The classic and most studied signaling pathway is EGFR pathway since both COX-2 and PGE_2_ are involved in the proliferation, migration and invasion of human colon carcinoma cells through EGFR [22, 23]. Combining EGFR tyrosine kinase inhibitor (erlotinib) with COX-2 inhibitor (celecoxib) can inhibit the tumor cells proliferation of head and neck cancer cell lines and the tumor growth of nude mouse xenograft models compared with either single agent [24]. Moveover, the biomarker expression (antigen Ki67, phosphorylated S6 and CD34) of head and neck cancer is decreased in 11 cancer patients received the combined treatment with erlotinib and celecoxib [24]. PGE_2_ can also trans-activate peroxisome proliferator-activated receptor β/δ (PPAR β/δ) via phosphatidylinositol 3-kinase/protein kinase B (PI3K/Akt) signaling to promote cell survival of intestinal adenoma [25]. In the mice model, PGE_2_ can stimulate tumor growth of intestinal adenoma in *Apc*^*min*^ mice, but not in *Apc*^*min*^ mice lacking PPAR β/δ [25]. As a nuclear transcription factor, PPAR β/δ binds as heterodimers with a retinoid X receptor (RXR) for transcription initiation [26], and the natural ligands for PPAR β/δ include fatty acid and PGE_2_ [27]. Additionally, PGE_2_ activates Ras-MAPK cascade and high expression of PGE_2_ can induce COX-2 expression in intestinal adenomas [28]. Studies concerning the COX-2-PGE_2_-EPs signaling pathway are limited in gynecological cancers compared with that in gastrointestinal cancer and breast cancer [29].

## Endometrial cancer

Endometrial cancer (EC) is the most common gynecological malignancy in developed countries, including the United States, Canada and Western Europe [30]. There estimated to be more than 61,000 new cases of EC and over 12,000 deaths in the United States according to the 2019 cancer statistics [31]. The main risk factor for EC is exposure to endogenous and exogenous estrogens, which is linked to obesity, diabetes, early age at menarche, null parity, late menopause and use of tamoxifen [32]. EC is classified into two subtypes: type I and type II. Type I is the most common subtype, and it is low-grade, endometrioid, diploid, hormone-receptor-positive endometrial cancer with a good prognosis [32]. By contrast, type II EC is high-grade, non-endometrioid, aneuploidy, hormone-receptor-negative, *TP53*-mutated with a poor prognosis and a higher risk of metastasis [32].

### COX-2 and endometrial cancer

COX-2 is expressed in the cytoplasm of normal proliferative glandular epithelium and endometrial cancer cells [33]. The mRNA level of COX-2 is elevated in 51 cancerous endometria compared with 16 normal endometria [34]. COX-2 is proved to be a negative predictor of disease relapse for EC patients in the univariate analysis. COX-2 plays a key role not only in the maintenance of the endometrium during the menstrual cycle but also in EC carcinogenesis [35]. COX-2 overexpression increases angiogenesis, migration, invasiveness and tumor-induced immmuno-suppression, as well as prevents apoptosis [35]. A combined treatment with celecoxib (a COX-2 inhibitor) and rapamycin (a mammalian target of rapamycin complex 1 inhibitor, a mTORC1 inhibitor) reduces EC progression in mouse models of EC and human EC cell lines [36]. Brasky et al. demonstrated that treatment of aspirin could reduce the risk of EC, especially in estrogen-mediated cases by analyzing 22,268 female Americans after up to 10 years [37]. In clinical studies, the correlation of COX-2 expression and EC patients’ prognosis still remains conflicting [38, 39]. PGE_2_ is associated with both endometrial functions and disorders. Ke et al. found that prostaglandin E synthase 2 (PTGES2) is upregulated in the 119 endometrial cancer tissues compared with 50 normal endometria, and PTGES2 is associated with the endometrial carcinoma stage, grade and the depth of myometrial invasion [40].

### EPs expression in endometrial cancer

**Fig. 2:**
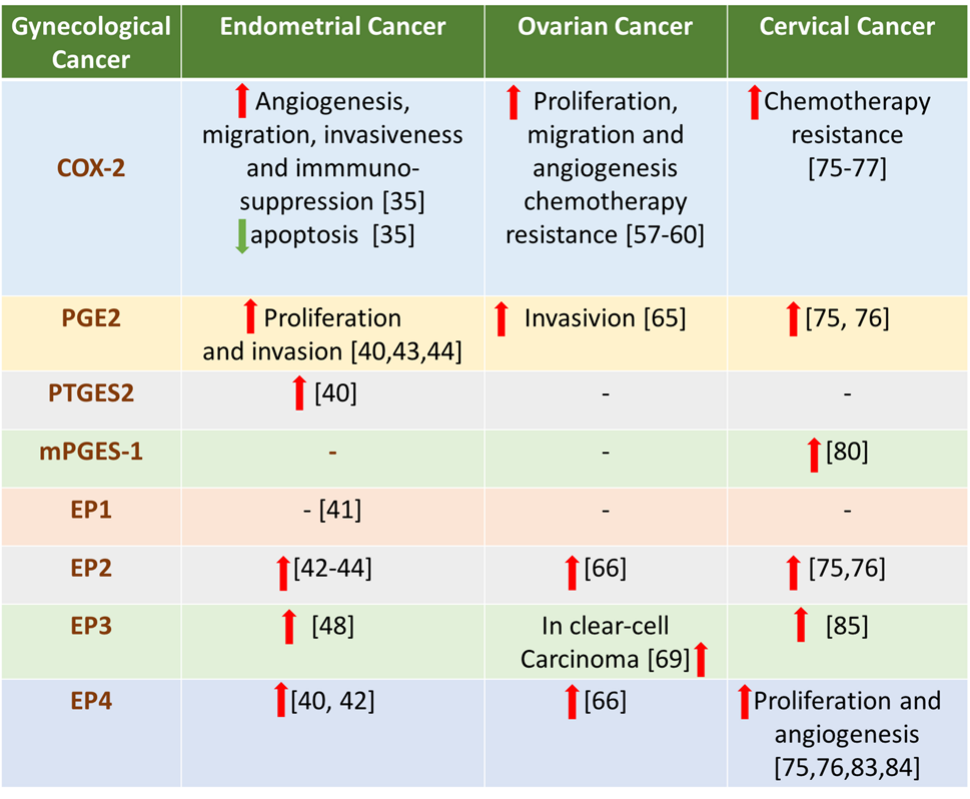
Overview the expression of COX-2-PGE2-EPs signaling in gynecological malignancies (endometrial cancer, ovarian cancer and cervical cancer). Red arrow: upregulation; green arrow:downregulation; -: uncertain

Zhu et al. suggested that six patients with higher EP1 staining survived after seven years follow-up, although EP1 expression was not correlated to progression-free survival or overall survival of endometrial cancer patients [41]. More recruited EC patients’ samples might further prove the connection of EP1 expression and EC (Fig. 2). The biosynthesis of EP2, EP4 and cAMP are significantly elevated in response to PGE_2_ in endometrial adenocarcinoma tissues compared with normal endometria by quantitative PCR [42]. PGE_2_ stimulates vascular endothelial growth factor (VEGF) expression in Ishikawa cells (a human endometrial adenocarcinoma cell line) via EP2-cAMP-mediated transactivation of the epidermal growth factor receptor (EGFR) and extracellular signal-regulated kinases 1/2 (ERK1/2) pathways [43]. Battersby and his colleagues proved that PGE_2_ upregulates the expression of fibroblast growth factor 2 (FGF2) via the EP2 receptor in a cAMP-, c-Src-, epidermal growth factor receptor (EGFR)- and extracellular signal-regulated kinase (ERK)-dependent manner in Ishikawa cells [44]. FGF2 is a potent mitogenic and angiogenic factor, causing adenocarcinoma cell proliferation in nude mice transplanted subcutaneously with endometrial adenocarcinoma [45]. PGE_2_ can enhance proliferation and invasion of two human endometrial cancer cells (Ishikawa and HEC-1B) by stimulating EP4 receptor and small ubiquitin-like modifier-1 (SUMO-1) via the Wnt/β-catenin signaling pathway [40].

The proliferation and angiogenesis of implanted tumor can be directly inhibited in *EP3*^−/−^ mice, as well as suppressed by an EP3 antagonist (ONO-AE3–240) in wild-type mice [46]. EP3 mRNA is expressed abundantly in the uterus [47]. The latest study by our research group demonstrated that high expression of EP3 correlates with poor progression-free survival and overall survival in endometrial carcinoma [48]. Our group also proved that L-798,106 (a specific EP3 antagonist) induces the expression of estrogen receptor β and inhibits the activity of Ras, leading to decreased proliferation and migration of RL95-2 cells [48]. Overexpression of estrogen receptor β (ERβ) inhibits proliferation and invasion of tumor cells in breast cancer and endometrium [49, 50].

## Ovarian cancer

Ovarian cancer ranks the fifth as a cause of neoplastic death among women worldwide [30] and the first lethal gynecological malignancy [31]. There would be around 22,530 new cases and 13,980 deaths in the United States in 2019 [31]. Incidence rates are highest in more developed regions, with rates of more than 7.5 per 100,000 [30]. The overall 5-year survival rate of ovarian cancer is just approximately 30–40% [51]. The fundamental problem in treating ovarian cancer is that it is not easy to discover it at an early stage and accomplish complete curative resection. Ovarian cancer histological groups include type I epithelial, type II epithelial, germ cell, sex cord-stromal, other specific non-epithelial and non-specific tumors [52]. Among all the subtypes, type II epithelial tumors are the most common in Oceania, North America and Europe, while type I epithelial tumors are more common in Asia during 2005–2009 [52]. Type II epithelial tumors are associated with poorer survival than type I epithelial, germ cell and sex cord-stromal tumors [52].

### COX and ovarian cancer

*COX-2*^*−/−*^ female mice show defective ovulation, fertilization and implantation [53]. The mRNA expressions of COX-1, COX-2, EP2 and EP4 are detected in both granulosa and cumulus cells in mice periovulatory follicles during superovulation [54]. COX-1, COX-2, mPGES-1, EP1 and EP2 are expressed predominantly in epithelial cells of human epithelial ovarian cancer [55]. Kino et al. (2005) believed that COX-1 is the primary enzyme for producing PGE_2_ instead of COX-2 in ovarian cancer cells. Because the elevated expression of COX-1 instead of COX-2 was detected in 22 ovarian cancer tissues compared with that in normal cases [56], and SC-560 (a COX-1 inhibitor) can suppress the production of PGE_2_ in three ovarian cancer cell lines while NS-398 and rofecoxib (COX-2 inhibitors) can not [56]. However, the latest meta-analysis (2017) suggested that COX-2 expression is correlated with FIGO stage, histological type and patients’ age and the high expression of COX-2 is associated with reduced OS and DFS of ovarian cancer patients [57]. In addition, high expression of COX-2 is associated with a shorter progression time and overall survival time in the patients who firstly underwent explorative laparotomy and then received chemotherapy [58]. It implies that COX-2 is also correlated with chemotherapy resistance.

For in vitro studies, COX-2 can stimulate the proliferation, migration and angiogenesis of ovarian cancer cells. COX-2 enhances proliferation and migration of human ovarian cancer CAOV-3 cells mainly through the activation of phosphatidylinositol 3-kinase/protein kinase B (PI3-k/Akt) pathway [59]. By analyzing the epithelial ovarian cancer (EOC) tissues and EOC cell lines (MDAH2274 and SKOV3), Uddin and his colleagues demonstrated that COX-2 modulates cell growth and apoptosis also through PI3K/AKT signaling pathway in EOC [60]. Furthermore, Uddin et al. proved that COX-2 could be a potential therapeutic target in EOC because treatment of xenografts together with aspirin can inhibit tumor growth in nude mice through decreasing the expression of COX-2 and Akt [60]. COX-2 protein levels correlate with VEGF protein levels and microvessel counts in ovarian carcinoma [61].

The expression of COX-2 is regulated by various cytokines in ovarian cancer cells, such EGF, vitamin D, IL-1β. EGF induces the production of both COX-2 and PGE_2_ via the activation of the PI3K/Akt signaling pathway, resulting in an invasion of SKOV3 and OVCAR5 cells (two human ovarian cancer cell lines) [62]. A reduced level of vitamin D receptor (VDR) and an induced level of COX-2, 15-PGDH and PGE_2_ are found in the serum of ovarian cancer patients older than 45 years [63], suggesting an interaction between PG and vitamin D-metabolism in ovarian cancer. The mRNA and protein expression of COX-2 can be stimulated by IL-1β and phorbol ester (TPA) in OVCAR-3 cells and by TPA in CAOV-3 cells [64].

### EPs and ovarian cancer

PGE_2_ can induce cell invasiveness via increasing the expression of MMP-2 and MMP-9 in two human ovarian cancer cell lines (CaOV-3 and SKOV-3) [65]. PGE_2_ stimulates the VEGF production in HEY ovarian cancer cells mainly via activating EP2 and EP4 receptors, which can be reversed by AH23848 (an antagonist of both EP2 and EP4) [66]. In addition, PGE_2_-induced EP4 receptor signaling induces MMP production and ovarian cancer cell invasion through Src-mediated EGFR transactivation [66]. EP2 plays a vital role in the process of ovulation and fertilization because cumulus expansion becomes decreased in *EP2*^*−/−*^ mice [67]. By feeding hens 10% flaxseed-enriched or standard diet for four years, Eilati et al*.* proved a decreased expression of COX-2 and PGE_2_, as well as a reduction in ovarian cancer severity and incidence [68]. Our research group (2019) has recently demonstrated that EP3 expression was higher in clear-cell carcinoma compared to three other histological subtypes of ovarian cancer [69]. Moreover, EP3 negative patients showed longer overall survival in a subgroup with negative expression of a tumor-specific epitope of tumor-associated epithelial mucin 1 (MUC1) [69].

## Cervical cancer

Cervical cancer is the fourth most common cancer and the fourth most common cause of cancer-related death among women worldwide [70]. The incidence and prevalence of cervical cancer are higher in the developing countries than in the developed countries owing to a lack of screening, availability of vaccine, and awareness of HPV infections [71]. In the United States in 2019, there were an estimated 13,170 cases and 4250 deaths from cervical cancer [31]. In many developing countries, cervical cancer causes more than one-quarter of a million deaths per year [72]. The 5-year survival rate of cervical cancer is between 63–79% in China, Singapore, South Korea and Turkey, less than 25% in Gambia and Uganda [73]. The two main malignant epithelial cervical cancer types are the squamous cell carcinoma and the adenocarcinoma [74].

### COX-2 and cervical cancer

High expressions of COX-2 and PGE_2_ have been found in the cervical carcinoma [75, 76]. COX-2 is overexpressed in various types of cervical neoplasm such as cervical intraepithelial neoplasia (CIN), adenocarcinoma and squamous cell carcinoma, implying that COX-2 expression is highly associated with cervical carcinoma development and progression [10]. Many studies show that COX-2 contributes to carcinogenesis and progression of cervical cancer. High expression of COX-2 is related to poor overall survival (OS) and poor disease-free survival (DFS) in cervical cancer patients [77]. COX-2 is also associated with poor DFS in chemo-radiation subgroup, implying COX-2 is a chemo-radiation resistance predictive factor for cervical cancer [77]. The COX-2 expression is more frequently expressed in adenocarcinoma than in squamous cell carcinoma by immunohistochemistry [78]. The co-expression of COX-2 and thymidine phosphorylase (TP) is related to poor 5-year disease-free and overall survival rates, suggesting that the combination of COX-2 and TP is a prognosticator for squamous cell carcinoma of the cervical cancer [79].

The expression of mPGES-1 is higher in squamous intraepithelial lesions and carcinoma of the uterine cervix compared with the normal cervical epithelium [80]. Radilova et al. found that COX-1 is also coupled with mPGES-1 for co-regulating PGE_2_ synthesis in human cervix cancer cells [81]. Dimethylcelecoxib (a non-COX-2 inhibitor) inhibits the early growth response protein 1 (EGR1) and transcription of mGPES-1 via an enhanced complex of NF-κB and histone deacetylase 1 (HDAC1) that binds to the EGR1 promoter in Hela cells [82].

### EPs and cervical cancer

Sales et al. reported that the syntheses of COX-2, PGE_2_, EP2, EP4 and cyclic adenosine monophosphate (cAMP) are up-regulated in cervical cancer tissue compared to that in the healthy cervix, suggesting that PGE_2_ may regulate neoplastic cell function via the EP2/EP4 receptors [75]. Sales and his colleagues further in 2002 proved that PGE_2_ could induce the expression of COX-2, EP4 and cAMP in Hela cells which were transiently transfected with EP2 or EP4 cDNA [76]. However, this research did not detect whether the expression of cAMP would be decreased after knocking out or knocking down the expression of EP2 or EP4. Kurt et al. observed that rapid accumulation of cAMP is produced in Hela cells after being stimulated with PGE_2_, which is mediated via the cAMP-linked EP2/EP4 receptors [76]. Both studies imply that PGE_2_ regulates the function of cervical cancer cells mainly via cAMP-linked EP2/EP4 signaling pathway.

Jung-Min et al. showed an increased expression of EP4 in 52 cervical cancer tissues compared with four healthy controls by immunohistochemistry [83]. This study also demonstrated that HPV16 E5 upregulated the activity of PGE_2_-EP4-cAMP signaling pathways by inducing the binding of cyclic adenosine monophosphate response element-binding protein (CREB) to a variant CRE site in the promoter of the human EP4 gene [83]. EP4 plays a role in the proliferation and angiogenesis of cervical cancer cells since GW627368X (a highly selective EP4 antagonist) inhibits the proliferation and angiogenesis of cervical carcinoma by blocking EP4/EGFR signaling pathway in cervical cancer cell lines (HeLa, SiHa and ME 180) and suppresses the tumor size in xenograft mice model [84].

Our group recently proved that overexpression of EP3 in cervical cancer patients is associated with impaired prognosis in overall survival rates when evaluating 250 cervical cancer patients with immunohistochemistry [85]. As an independent prognosticator for cervical carcinoma, the EP3 receptor is also significantly correlated with lymph node stage and FIGO stage [85]. However, the pathological mechanism of how EP3 signaling regulates in cervical cancer is still unclear.

## Drug targeting of COX-2-PGE_2_-EPs signaling

Chemoprevention has long been recognized as an important prophylactic strategy to reduce the burden of cancer on the health care system. In addition, nonsteroidal anti-inflammatory drugs (NSAIDs) as chemoprevention chemicals have been proved to reduce the risk of several cancers in human, such as gastrointestinal cancer, breast cancer, prostate cancer, lung cancer and skin cancer [86]. Nan et al. (2015) found that regular use of aspirin or NSAIDs is linked to lower risk of colorectal cancer compared with no regular use after analyzing 8634 colorectal cancer cases and 8553 matched controls between 1976 and 2011 [87]. Long-term use of COX inhibitors in humans leads to a 50% decrease in risk for colorectal cancer [25]. A meta-analysis by Banndrup et al. suggested the risk of invasive ovarian cancer is significantly reduced with the use of aspirin [88].

NSAIDs include aspirin, ibuprofen and naproxen, and act by inhibiting both COX-1 and COX-2. These unspecific inhibitors cause many adverse effects, such as gastrointestinal ulcers and bleeds, heart attack and kidney disease [89]. Selective COX-2 inhibitors (COXibs) has been successfully documented and showed less toxicity to gastrointestinal tract as compared to traditional NSAIDs [90]. However, the long-term use of COX-2 selective inhibitors still has other side effects. The adverse effects of COX-2 selective and non-selective inhibitors are summarized in the latest review by Rayar et al. including myocardial infarction, hypertension, stroke, reduced glomerular filtration rate and renal plasma flow, acute renal failure, acute interstitial nephritis, inhibition of ulcer healing, hepatic complications, allergy, fatal skin reaction, depression, delayed follicular rupture and so on [91]. Therefore, further exploration of novel anti-inflammation targets is needed.

Clinical studies show elevated levels of mPGES-1 are identified in colon, lung, stomach, pancreas, cervix, prostate, papillary thyroid carcinoma, head and neck squmaous carcinoma and brain tumors, suggesting mPGES-1 inhibitors might be a potential chemopreventive agent [16]. However, a limited number of compounds that inhibiting mPGES-1 has not been successfully developed as anti-cancer agents, such as celecoxib, MF-63, NS-398, MK-866 and triclosan [16].

In recent years, extensive efforts have been made into elucidating the function of PGE_2_ and the EP receptors in health and carcinogenesis with the aim of exploring promising targets and selective inhibitors as a novel therapy. Many researchers have found the strong correlation of EP2/EP4 receptors with colon cancer, skin cancer, mucosa cancer of the pharynx and the esophagus, prostate cancer, urothelial cancer and non-small cell lung cancer [18]. The EP4 promotes migration, invasion, angiogenesis and lymphangiogenesis of mammary tumor cells [92]. EP4 receptor is responsible for the PGE_2_-induced colorectal tumor cell proliferation and morphogenic changes via PI3k/Akt signaling pathway [93]. Mice are not able to have inflammatory responses to PGE_2_, IL-1β or lipopolysaccharide when lacking the EP3 receptor, but not EP1, EP2 or EP4 receptor [94]. Deletion or inhibition of EP3 receptors could ameliorate the neuronal apoptosis in the ischemic cortex in EP3 knock-out mice or EP3 antagonist-treated mice compared with wild-type mice or vehicle-treated mice, respectively. It suggests that EP3 is involved in the inflammatory and apoptotic reactions during stroke injury [95]. The investigations concerning EP receptors in gynecological cancers highlight the potential advantage of combining COX enzyme inhibitors with EP receptor antagonists as therapeutic agents in gynecological cancers.

Wang et al. proposed the possible PGE_2_ downstream targets that might also serve as promising specific chemopreventive agents for cancer prevention and treatment, which include angiogenic factors (VEGF, bFGF), anti-apoptotic factors (Bcal-2), chemokines (MIP-1α, MIP-1β, RANTES, CXCR4) and their receptors, and immunosuppressive mediators [7].

## Conclusions

Studies show that COX-2 is a negative predicator of EC, ovarian cancer and cervical cancer, contributing to carcinogenesis and progression of gynecological cancer. The expression of mPGES-1 is highly expressed in human epithelial ovarian cancer and cervical cancer, while PTGES2 is associated with the endometrial carcinoma carcinogenesis. Many studies focus the function of cAMP-linked EP2/EP4 signaling pathway in EC, ovarian cancer and cervical cancer, while roles of EP1 and EP3 have not been completely clarified. Further investigations concerning EP receptors in gynecological cancers are necessary. EPs may represent novel and specific anti-inflammation targets for gynecological cancer chemoprevention. It might highlight the potential advantage of combining COX enzyme inhibitors with EP receptor antagonists as therapeutic agents in gynecological cancer.
